# A Novel Synthesis of the Efficient Anti-Coccidial Drug Halofuginone Hydrobromide

**DOI:** 10.3390/molecules22071086

**Published:** 2017-06-30

**Authors:** Junren Zhang, Qizheng Yao, Zuliang Liu

**Affiliations:** 1 School of Chemical Engineering, Nanjing University of Science and Technology, Nanjing 210094, China; zjr1108@njau.edu.cn; 2 Laboratory of Veterinary Pharmacology and Toxicology, College of Veterinary Medicine, Nanjing Agricultural University, Nanjing 210095, China; 3 School of Pharmacy, China Pharmaceutical University, Nanjing 210009, China; qz_yao@163.com

**Keywords:** febrifugine, halofuginone hydrobromide, asymmetric synthesis, 4(3*H*)-quinazolinone, piperidine

## Abstract

*Background*: Halofuginone hydrobromide (**1**) is recognized as an effective drug against several species of *Eimeria* (E.) in poultry. In this paper, we describe a convenient and low cost preparation method for the compound, as well as primary validation of its activity. *Methods*: First, 7-bromo-6-chloroquinazolin-4(3*H*)-one (**2**) was prepared from *m*-chlorotoluene by a conventional process, and then chloroacetone was creatively introduced in two steps. Finally, halofuginone hydrobromide (**1**) was obtained from 7-bromo-6-chloro-3-(3-cholroacetonyl) quinazolin-4(3*H*)-one (**4**) by a four-step reaction sequence including condensation, cyclization, deprotection and isomerization. The structures of the relative intermediates and target compound were characterized by melting point, IR, MS and ^1^H-NMR. Besides, the protective effect of compound **1**-supplemented chicken diet at doses of 6, 3 and 1.5 mg per 1 kg were evaluated on chickens infected with *E. tenella*, by reduction in mortality, weight loss, fecal oocyst excretion and gut pathology, respectively. *Results*: Halofuginone hydrobromide (**1**) was prepared successfully by and improved and innovative method based on traditional research. Moreover, the synthesized halofuginone hydrobromide significantly exhibited an anti-coccidial property. *Conclusions*: The fruitful work described in this Communication has resulted in halofuginone hydrobromide, which has a good pharmaceutical development prospects, becoming more available for large-scale production.

## 1. Introduction

**Figure 1:**
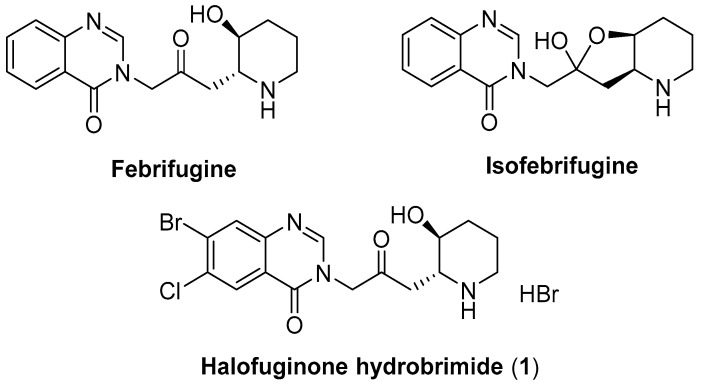
Structures of febrifugine, isofebrifugine, and halofuginone hydrobrimide (**1**).

Febrifugine and isofebrifugine (Figure 1), were first isolated from leaves of *Dichroa Febrifuga Lour* in 1948, and two years later, their skeletal structures were reported [1]. After more than 40 years of research, determination of the absolute configuration of the two natural products was achieved (febrifugine: 2*R*,3*S*; isofebrifugine: 2*S*,3*S*) [2]. In 1967, a derivative of febrifugine named halofuginone hydrobromide (**1**) was first synthesized by American Cyanamid Company, which later transferred this product to Roussel Uclaf S. A. who marketed it in a premix named “Stenorol” [3]. Halofuginone hydrobromide has been proved to possess broad pharmacological functions, such as anti-coccidial [4,5,6], anti-fibrosis [7,8,9], anti-tumor [10,11,12] and growth-promoting activities [13]. In clinic application, addition at a dose of 3 mg·kg^−1^ in animal feed exhibit great effects against almost all types of coccidia [14,15,16].

**Figure 2:**
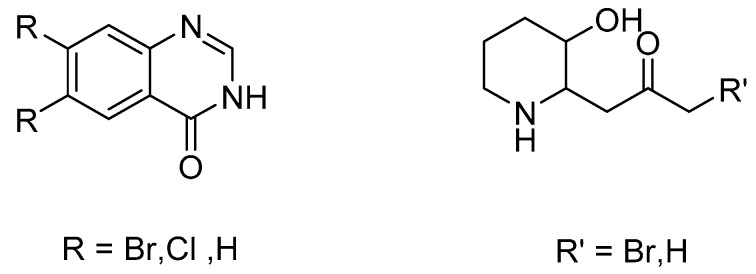
4(3*H*)-Quinazolinone (**L**) and piperidine (**R**) derivative.

Many other researchers have focused on the preparation of halofuginone and relevant compounds, and they finally figured out the key point was to generate two intermediates involving substituted 4(3*H*)-quinazolinone and piperidine moieties (Figure 2) [17].

**Scheme 1:**
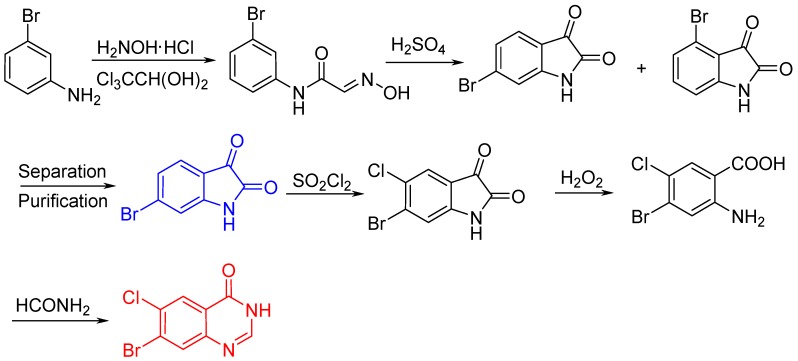
Route A: Synthesis of 7-bromo-6-chloroquinazolin-4(3*H*)-one: The separation and purification of bromo-substituted 2,3-indolinedione proved hard, leading to low total recovery.

**Scheme 2:**
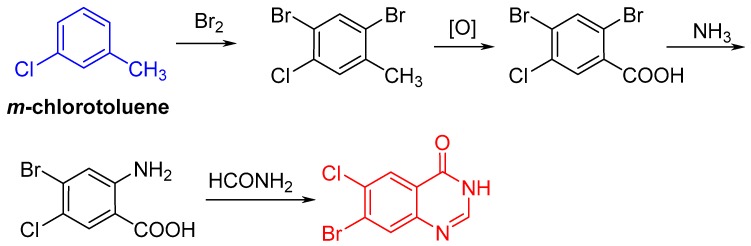
Route B: synthesis of 7-bromo-6-chloroquinazolin-4(3*H*)-one: by choosing 3-chlorotoluene as the starting material, the intermediate was produced with a reduced number of reaction steps and cost.

With regard to the 4(3*H*)-quinazolinone fragment, the preparation is quite mature as so far there have be a lot of studies reporting synthesis methods for this target. Route A (Scheme 1) would be the most classical method, and many other reports came up with new syntheses based on Route A by changing the starting material or reaction conditions [18]. On the other hand, route B (Scheme 2) which features a short process, mild conditions and easy treatment of the three waste streams in particular, was taken as reference to design a rational technological process [19,20,21].

**Scheme 3:**
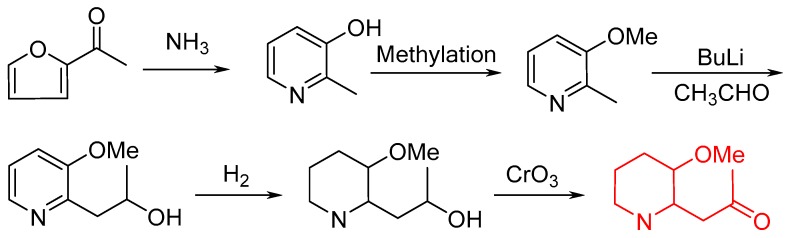
Synthesis of 2-acetonyl-3-methoxyl piperidine involving the application of pyridine derivatives.

The preparation of piperidine analogues was the most expensive part, and deemed to be the real difficulty to produce halofuginone as well. So far, a lot of methods have been reported: The method illustrated in Scheme 3 was originally developed by Baker’s team [18,22,23,24,25,26]. Afterwards, the method was optimized a bit by Barringer et al., via changing reagents and catalyst, which successfully improved the yield by reducing the number of steps but without lowering the cost. Referring to Baker’s route, Wu’s group invented a different approach to 2-acetonyl-3-methoxypiperidine using 3-hydroxyl pyridine as starting material; before this, Takeuchi et al., had already synthesized piperidine derivatives from 3-hydroxypyridine [27]. However, all these synthesis involved a reduction of the pyridine ring, which led to high costs and considerable pressure for environmental reasons.

**Scheme 4:**
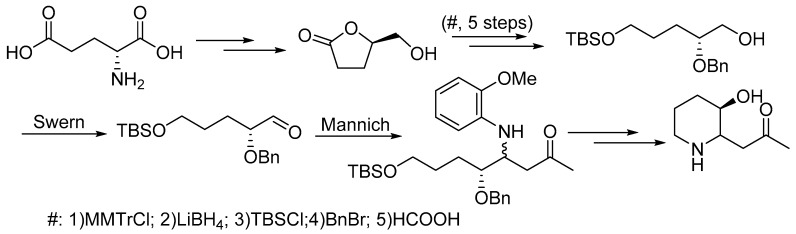
First asymmetric synthesis of the piperidine ring.

In addition, some other researchers studied different ways to prepare the piperidine ring. In 1999, Kobayashi et al., were the first to successfully prepare the desired piperidine intermediate (Scheme 4), resulting in further confirmation of the relative configuration of febrifugine and making possible the asymmetric synthesis of febrifugine analogues [2].

**Scheme 5:**
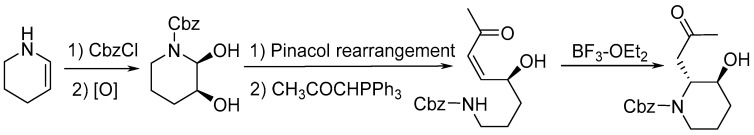
The shortest preparation of the piperidine derivative.

However, the previous methods required complex operations and too many steps, whereas there was one method that should be mentioned (Scheme 5), which might be useful for industrial production because of its high yield and very few reaction steps [28]. Nevertheless, the cost of production was always very high, so there is a strong demand for a new simple approach to produce the piperidine intermediate for halofuginone at lower cost.

In the present study, a new expedient total synthesis of halofuginone hydrobromide was developed. In addition, its anti-coccidial activity in chickens, as evidenced by survival rate, body weight loss, oocyst shedding and intestine pathology, was examined [5,6].

## 2. Results and Discussion

### 2.1. Synthesis of Halofuginone Hydrobromide

**Scheme 6:**
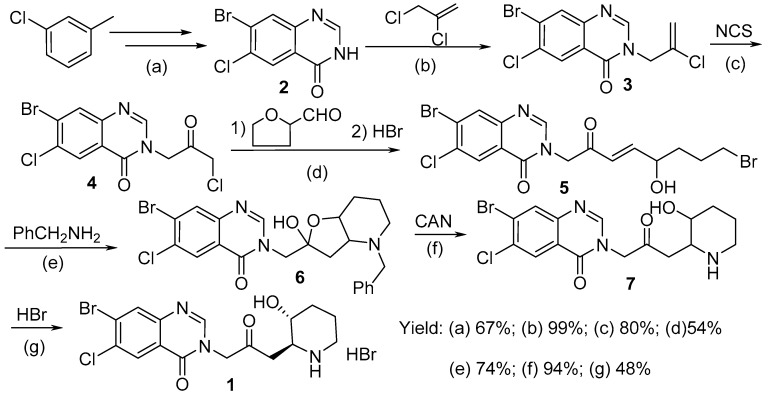
Total synthesis process to halofuginone hydrobromide **1**.

Our synthetic route to halofuginone hydrobromide is described in Scheme 6. Firstly, compound **2** was prepared from 3-chlorotoluene by the optimized method of Route B (Scheme 2) under improved conditions, which proved our most difficult and challenging task.

**Scheme 7:**
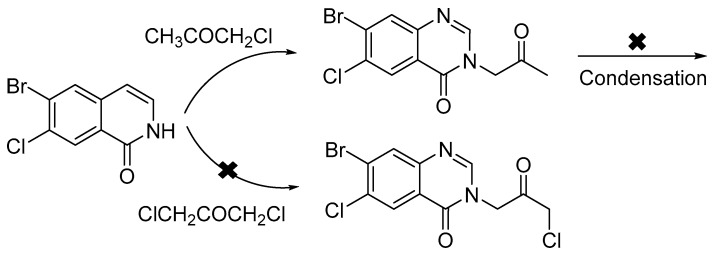
Two unsatisfactory routes to substituted products.

Initially, we produced 3-acetonyl-6-chloro-7-bromoquinazolin-4(3*H*)-one using 1-chloroacetone (Scheme 7). Then we tried to carry out the next condensation, but failed to obtain good yields of the target compound even under various optimized conditions. We then attempted to introduce **2** into 1,3-dichloroacetone by single substitution, but failed too. Finally, chloroacetonylation was separated into two parts: condensation of **2** with 2,3-dichloropropylene and subsequent addition with NCS and acidic hydrolysis, to afford a convenient conversion of **2** to **4** in satisfactory yield. Then, condensation of **4** with tetrahydrofuran-2-carbaldehyde in ethanol was performed as to simultaneously introduce the hydroxyl, as well as a double bond into **4**, followed by direct bromination to give **5**. With regards to the condensation, the influences of different catalysts and solvents were investigated (Table 1). As a result, potassium *tert*-butoxide (KTB) was beneficial to the catalysis, while ethanol was the most suitable solvent even for large scale production. Compound **7** was not formed by direct cyclization with NH_3_ gas, so **5** was first reacted with benzylamine to give **6**, which could be quickly carried out at R.T. and then **7** was successfully obtained by debenzylation with CAN. Finally, the *cis*- and *trans*-isomer of halofuginone hydrobromide were produced, and *trans*-isomer (**1**) was separated and purified thanks to of their different solubility in ethanol, which was verified by NMR data referring to Uesato et al. [29].

### 2.2. Evaluation on Antioccidial Activity of Halofuginone Hydrobromide (Hh) in Chickens

After acclimatization for 20 days on standard diet alone, 50 individual chickens with similar body weights were chosen, and were fed standard diet alone (I, II) or supplemented with different doses (III, IV, V) of compound **1**. One day later, animals (II~V) were infected by oral administration of 100,000 sporulated oocysts. Animals of the infected unmedicated group (Group II) showed obvious clinical symptoms from 4 days post-inoculation, mainly as follows: general malaise, reduced ingestion, emerging bloody stools, and even death. In contrast, all animals of the uninfected unmedicated group (Group I) were very healthy, with no negative symptoms being observed. On day 7 post-infection, there was no difference in the body weight of the chickens in Group IV (Hh3) in comparison with Group I (UI control), In contrast, the body weight of chickens in Group III and Group V were significantly different from Group I (UI control). Furthermore, the body weight of chickens in the three infected medicated groups (III–V) was much higher than Group II. Overall, halofuginone hydrobromide significantly ameliorated the reduced weight gain caused by *E. tenella* to a greater degree, and even as slight weight-gain effect was observed, which was accelerated by the medium dose (0.3%). No fecal oocysts were detected in the uninfected unmedicated controls. Only a few fecal oocysts were detected in the three medicated groups, and the relative reduction rate of oocysts (RORR) in each group (II–V) was 0%, 98.8%, 95.2%, 86.3%, respectively. It turned out that halofuginone could strongly inhibit reproduction of coccidia. All the chickens in each group were sacrificed on day 7 post-infection and their ceca were removed. Gross lesion scores were obtained as described previously. The uninfected unmedicated control chickens (Group I) had no lesions in the ceca (score = 0). In contrast, *E. tenella* caused more gross cecal lesions in the gut of unmedicated chickens 7 days post-infection, as evidenced by a lesion score close to 3.2 (Group II). Halofuginone at different doses (0.6%, 0.3% and 0.15%) significantly diminished the cecal damage in infected chickens (Groups III–V) as shown by the gross lesion scores of 0–1.1 (0, 0.2, and 1.1). Comprehensive results are listed below (Table 2).

## 3. Experimental Section 

### 3.1. General Information

Every reaction was monitored, and the endpoint was checked, by TLC performed on GF-254 silica gel plates (Yantai Derxin Biological Technology, Yantai, China) with visualization by UV light. Melting points were measured on an YRT-3 temperature apparatus (Tianjin Jing Tuo Instrument Technology, Tianjin, China) and are uncorrected. IR spectra were recorded on a VERTEX 80 instrument (Bruker Corporation (Beijing), Beijing, China)). Mass spectra were determined on a VG Auto Spec-3000 spectrometer (VG Instruments, Kuala Lumpur, Malaysia) and reported as *m*/*z*. The ^1^H-NMR spectral data were recorded on a Bruker Avance III HD spectrometer (300~900 MHz, Bruker Corporation (Beijing)), and chemical shifts were reported in ppm (δ) relative to TMS as an internal standard.

### 3.2. Synthesis

#### 3.2.1. Synthesis of 7-bromo-6-choroquinazolin-4(3*H*)-one (**2**)

To a mixture of 3-chlorotoluene (200 g, 1.58 mol), FeCl_3_ (9.4 g, 0.06 mol) and CH_2_Cl_2_ (1000 mL) stirred at 0~10 °C, Br_2_ (500 g, 3.12 mol) was added slowly, during which the produced gas was absorbed by 15% NaOH aqueous solution (2000 mL). After 1 h of continued vigorous stirring, water (100 mL) was added and the mixture was adjusted to pH 8.0~10.0 with 10% aqueous NaOH (200 mL). The organic layer was separated and concentrated to dryness under vacuum to give a solid (95.7%, m.p. 95~96 °C), 426 g of which was added to a mixture of water (2000 mL), pyridine (200 mL) and KOH (112 g, 2.0 mol) and heated. When the interior temperature reached 80 °C, KMnO_4_ (800 g, 5.0 mol) was added in portions, and the mixture was refluxed until complete consumption of the KMnO_4_. Then the solution was filtered, the precipitate was washed with 10% KOH a.q. (1000 mL). The filtrates were combined, and then vacuum evaporated to remove the pyridine and form a turbid solution, which needed to be filtered again. The fresh filtrate was acidified with concentrated HCl (360 mL) to pH 3~4. Then the precipitate was filtered, dried and crushed (80%, m.p. 170~171 °C). A portion (340 g) was dissolved in 25% NH_3_-H_2_O (1700 mL). To the solution, Cu_2_O was added in batches at 30~40 °C. After about 5 h of stirring, the solution without NH_3_ gas was diluted and acidified. Then the solid was filtered, washed, dried (yield 96%, m.p. 248~249 °C) and 250 g was dissolved in 1500 mL of formamide, maintained at 150~170 °C for 5 h. The solution was allowed to cool to R.T., and the precipitate was filtered and washed to give a white solid (92%, m.p. 306~307 °C). Thus, **2** was succesfully obtained in an overall yield of 67%. IR (KBr), ν (cm^−1^): 3188.6, 1643.4 (NH), 1680.3 (C=O); ESI-MS (*m*/*z*): 256.9 [M − H]^−^, 258.9 [M + 2 − H]^−^; ^1^H-NMR (300 MHz, DMSO-*d*_6_), δ (ppm): 12.50 (br, 1H), 8.15 (s, 1H), 8.14 (s, 1H), 8.05 (s, 1H).

#### 3.2.2. 7-Bromo-6-chloro-3-(2-chloropropenyl)quinazolin-4(3*H*)-one (**3**)

A mixture of compound **2** (100 g, 0.38 mol), 2,3-dichloropropylene (52 g, 0.47 mol), K_2_CO_3_ (83 g, 0.6 mol) and DMF (300 mL) was stirred for 1 h at 120 °C, and then maintained at 115~125 °C for another 7 h. Immediately after that, the reaction mixture was transferred into the mixture of ice and water to cool to 5 °C. After stirring for 1 h, the precipitate was filtered, washed, and extracted in ethyl acetate. The fresh organic phase was completely washed with saturated aqueous NaCl, and the organic layer was concentrated to dryness under vacuum to give **3** (127 g, 99%, m.p. 150~155 °C). IR (KBr), ν (cm^−1^): 3057.0, 907.6 (=CH_2_), 1685.1 (C=O); ESI-MS (*m*/*z*): 333.0 [M + H]^+^, 335.0 [M + 2 + H]^+^; ^1^H-NMR (400 MHz, DMSO-*d*_6_), δ (ppm): 8.44 (s, 1H), 8.24 (s, 1H), 8.15 (s, 1H), 5.5 (s, 2H), 4.8 (s, 2H). ^13^C-NMR (CDCl_3_), δ (ppm): 51.8, 117.4, 122.0, 127.8, 129.7, 132.8, 133.9, 134.7, 146.9, 147.1, 159.2.

#### 3.2.3. 7-Bromo-6-chloro-3-(3-chloroacetonyl)quinazalin-4(3*H*)-one (**4**)

To acetonitrile (500 mL) stirred at 20 °C, **3** (127 g, 0.38 mol) was added together with 5% aqueous HCl (20 mL) and then NCS (100 g, 0.75 mol) was added in batches at 20 ± 2 °C within 4 h. The solution was allowed to cool for 30 min, and the precipitate was filtered and dried to give 106 g of compound **4** (80%, m.p. 239~241 °C). IR (KBr), ν (cm^−1^): 2993.0, 2968.1, 2930.0, 1447.7 (CH_2_), 1735.9 (C=O, keto carbonyl group), 1667.1(C=O, acyl carbonyl group); ESI-MS (*m*/*z*): 349.0 [M + H]^+^, 351.0 [M + 2 + H]^+^; ^1^H-NMR (400 MHz, DMSO-*d*_6_), δ (ppm): 8.29 (s, 1H), 8.22 (s, 1H), 8.16 (s, 1H), 5.07 (s, 2H), 4.74 (s, 2H); ^13^C-NMR (DMSO-*d*_6_), δ (ppm): 47.7, 53.1, 122.2, 127.4, 129.0, 132.3, 132.9, 147.7, 150.0, 159.2, 196.5.

#### 3.2.4. 7-Bromo-6-chloro-3-(8-bromo-5-hydroxyl-2-oxo-3-octenyl) quinazolin-4(3*H*)-one (**5**)

A mixture of **4** (35 g, 0.1 mol), anhydrous ethanol (300 mL), KTB (15 g, 0.13 mol), and 2-furan-carboxaldehyde (12 g, 0.12 mol) was refluxed for 7 h. The mixture was initially cooled to 40 °C naturally, then quickly cooled to below 0 °C in ice-salt water. Upon first addition of hydrobromic ethanol (40 mL, 30%), the temperature went up to 5 °C automatically. The reaction mixture was kept at that temperarure for 2 h, then a by second addition of hydrobromic ethanol (13 mL), maintained at 50 °C for 5 h. As soon as the mixture cooled to 0 °C, NaHCO_3_ (20 g) was added in batches, and after stirring for 30 min, the mixture was redispersed by addition of water (50 mL). Then, the mixxed solution was vacuum evaporated to recover the ethanol. Finally, the precipitate was filtered and dried to give crude product (40 g), which needed recrystallization to achieve purer **5** (26 g, 54%, m.p. 123~124 °C). IR (KBr), ν (cm^−^^1^): 3291.5 (OH), 2983.5–2843.0, 725.2 (CH_2_), 1698.1 (C=O, ketocarbonyl group), 1681.1 (C=O, acyl carbonyl group); ESI-MS (*m*/*z*): 474.9 [M − H]^−^, 476.9 [M + 2 − H]^−^; ^1^H-NMR (300 MHz, DMSO-*d*_6_), δ (ppm): 8.33 (s, 1H), 8.21 (s, 1H), 8.15 (s, 1H), 7.06–7.13 (dd, 1H), 6.38–6.43 (d, 1H), 5.18 (s,2H), 4.31 (m, 1H), 3.57 (t, 2H), 1.91 (m, 2H), 1.64 (m, 2H); ^13^C-NMR (DMSO-*d*_6_), δ (ppm): 29.0, 35.0, 35.6, 53.5, 69.1, 122.3, 125.2, 127.4, 128.8, 132.2, 132.9, 147.8, 150.4, 152.7, 159.2, 192.7

#### 3.2.5. 7-Bromo-6-chloro-3-[(4-benzyl-2-hydroxyl-octahydrofuran[3,2-*b*]pyridine-2)methyl]quinazol-in-4(3*H*)-one (**6**)

Compound **5** (50 g, 0.11 mol) was dissolved in CH_2_Cl_2_ (350 mL) and the temperature was controlled at 15~20 °C. To the solution, benzylamine (14.6 g, 0.14 mol) was added dropwise within 30 min, continuous stirring for 2 h. CH_2_Cl_2_ was recovered and the residue was dissolved in ethanol with refluxing for 30 min, then cooled, and the precipitate that formed was filtered to provide **6** as a light yellow powder (40.9 g, 74%, m.p. 183~185 °C). IR (KBr), ν (cm^−1^): 2961.1–2837.7 (CH_2_), 1680.5 (C=O, acyl carbonyl group), 1600, 1444.4 (benzene ring); ESI-MS (*m*/*z*): 504.1, [M + H]^+^, 506.1 [M + 2 + H]^+^, 502.0, [M − H]^−^, 504.1 [M + 2 − H]^−^; ^1^H-NMR (300 MHz, DMSO-*d*_6_), δ (ppm): 8.33 (s, 1H), 8.24 (s, 1H), 8.11 (s, 1H), 7.32–7.17 (m, 5H), 4.27–4.32 (d, 1H), 4.10–4.15 (d, 1H), 3.92–3.99 (m, 2H), 3.05–3.10 (d, 1H), 2.79 (s, 1H), 2.62–2.66 (d, 1H), 2.27–2.31 (d, 1H), 1.97–1.99 (d, 1H), 1.93–1.95 (d, 1H), 1.83–1.88 (d, 1H), 1.56–1.60 (d, 1H), 1.49–1.53 (d, 1H), 1.41 (d, 1H), 6.69 (s, 1H); ^13^C-NMR (CDCl_3_), δ (ppm): 19.9, 26.9,40.5, 50.0, 50.4, 59.3, 62.3, 82.9, 104.7, 122.0,127.6, 127.8, 128.6,128.6, 129.2, 129.2, 129.4, 132.7, 133.4, 136.6, 147.1, 149.3, 160.1.

#### 3.2.6. 7-Bromo-6-chloro-3-[3-(3-hydroxyl-2-piperidyl)-2-oxopropyl]quinazolin-4(3*H*)-one (**7**)

To a mixture of ethanol and H_2_O (ethanol:H_2_O = 4:1, 375 mL), compound **6** (40 g, 0.08 mol) was added at 20–30 °C. Batchwise addition of ceric ammonium nitrate (CAN) (92 g, 0.17 mol) was finished over 1 h. The reaction was continued for 8–9 h until the reaction endpoint was indicated by TLC. The mixture was adjusted to pH 8.0–9.0 with saturated aqueous Na_2_CO_3_ and then the product was extracted with CH_2_Cl_2_ (300 mL × 3). The combined organic solution was washed with saturated aqueous NaCl and then distilled. The white solid was recrystallized with ethanol, filtered, and dried to give 32.3 g of compound **7** in a yield of 94%; m.p. 215–217 °C. IR (KBr), ν (cm^−1^): 3416.5 (NH), 2974.9–2931.9 (CH_2_), 1715.8 (C=O, keto carbonyl group), 1679.7 (C=O, acyl carbonyl group); ESI-MS (*m*/*z*): 414.0 [M + H]^+^, 416.0 [M + 2 + H]^+^, 412.0, [M − H]^−^, 414.1 [M + 2 − H]^−^; ^1^H-NMR (300 MHz, DMSO-*d*_6_), δ (ppm): 8.25 (s, 1H), 8.23 (s, 1H), 8.17 (s, 1H),5.00 (s, 2H), 4.76–4.77 (d, 1H), 2.96–3.30 (dd, 2H), 2.50–2.51 (d, 1H), 2.45–2.48 (d, 1H), 2.36–2.44 (dd, 2H), 1.99(s, 1H), 1.88–1.91 (m, 1H), 1.56–1.59 (m, 1H), 1.30–1.40 (m, 1H), 1.17–1.27 (m, 1H); ^13^C-NMR (DMSO-*d*_6_), δ (ppm): 26.3, 39.4, 40.6, 46.1, 55.3, 60.6, 71.2, 122.3, 127.4, 128.8, 132.2, 132.9, 147.8, 150.2, 159.1, 203.9.

#### 3.2.7. (±)-*trans*-7-Bromo-6-chloro-3-[3-(3-hydroxy-2-piperidyl)acetonyl]quinazolin-4(3*H*)-one hydro-bromide (halofuginone hydrobromide, **1**)

Compound **7** (14 g, 0.034 mol) was dissolved in ethanol (300 mL) and stirred at 15–25 °C, then hydrobromic ethanol (10%, 33.2 g) was added dropwise within 30 min. After 1 h of stirring at R.T., the mixture was stirred at 60–70 °C for another 1 h. Then the solution was allowed to cool to 5–10 °C, and filtered to achieve the final pure product **1** (48%, 8.1 g, m.p. 245 (decomposed)). IR (KBr), ν (cm^−1^): 3327.3 (OH), 2943.1–2812.1(CH_2_), 1729.3 (C=O, keto carbonyl group), 1678.8 (C=O, acyl carbonyl group); ESI-MS (*m*/*z*): 414.0 [M + H]^+^, 416.0 [M + 2 + H]^+^, 412.0, [M − H]^−^, 414.0 [M + 2 − H]^−^; ^1^H-NMR (300 MHz, DMSO-*d*_6_), δ (ppm): 8.30 (s, 1H), 8.22 (s, 1H), 8.17 (s, 1H), 5.56 (d, 1H), 5.11 (s, 2H), 3.50–3.54 (m, 1H), 3.31–3.34 (m, 1H), 3.20–3.22 (m, 1H), 3.15 (m, 1H), 2.95–2.97 (m, 1H),2.87–2.89 (m, 1H), 1.92–1.96 (m, 1H), 1.80–1.91 (m, 1H), 1.52–1.71 (m, 1H), 1.41–1.51 (m, 1H), 8.69 (active hydrogen). ^13^C-NMR (DMSO-*d*_6_), δ (ppm): 20.2, 30.6, 39.5, 43.0, 54.4, 56.3, 66.7, 121.7, 126.8, 128.4, 131.8, 132.4, 147.2, 149.5, 158.6, 200.6.

### 3.3. Evaluation on the Antioccidial Activity of Halofuginone Hydrobromide in Chickens

Halofuginone is known worldwide as an efficient anticoccidial medicine. Therefore, the anticoccidial activity of our synthesized product needed to be verified as well. A chicken coccidiosis model was successfully induced by oral administration of sporulated oocysts (about 10^5^), indicating that the fecundity of *E. tenella* was strong enough. After acclimatization for 20 days on standard diet alone, 50 individual chickens with similar body weights were chosen, and were fed standard diet alone (I, II) or supplemented with different doses (III, IV, V) of halofuginone. One day later, all animals were infected. We monitored body weight of chickens with access to different diets after *Eimeria* infection. Body weight gain rate (BWGR) was calculated by the formula: BW on day 7 post-infection − BW on day 0 post-infection. To further determine the anti-coccidial effect, *Eimeria* oocysts in chicken feces, as an indicator of *Eimeria* multiplication, was evaluated. Fecal oocyst excretion of each group was collected from day 5 to day 7 post-infection and combined. The relative reduction rate of oocyst (RORR) was obtained by the formula: (OPG of Group II—OPG of each medicated group) ÷ OPG of Group II (OPG: oocyst per gram). All the chickens in each group were sacrificed on day 7 post-infection and their ceca were removed. Gross lesions in the ceca caused by *E. tenella* were scored based on five grades as described previously [30]. Finally, survival rate (SR) was recorded, contributing to calculation of ACI:

(1) $${\mathrm{ACI}}^{a}={\mathrm{RBWGR}}^{b}+{\mathrm{SR}}^{c}-{\mathrm{ALS}}^{d}\times 10-{\mathrm{OS}}^{e}$$

Notes: ^a^ Anticoccidial index (ACI): briefly, the efficiency of the drug was graded on the basis of the ACI value as follows: (1) high efficacy, >180; (2) moderate efficacy, 160~180; (3) low efficacy, 120~160; (4) invalid efficacy, <120. ^b^ Relative Body weight gain rate (RBWGR) was obtained by the formula: BWGR of each group ÷ BWGR of group I. ^c^ Survival rate (SR) was obtained by the formula: number of survival chickens in each group ÷ initial number of chickens. ^d^ Average lesion scores (ALS) was obtained by the formula: Gross lesion score ÷ numbers of chickens. ^e^ Oocyst score (OS) was obtained according to the standard described previously.

## 4. Conclusions

So far, there has been a lot of studies on developing synthesis for halofuginone, but all have failed to realize industrialization. To solve this problem, we developed the creative route for the total synthesis for halofuginone (overall yield 9.6%, 10 steps), of which efficient anticoccidial activity was evaluated and verified. Our method was characterized no use of harmful materials (except HBr) and no harsh conditions. In addition, we broke the traditional bottleneck (convergent synthesis) to achieve a great success by linear synthesis, reducing the cost by 70% [31]. However, many steps and time-costs still existed, which are the common drawbacks of linear synthesis. Aside from that, the final reaction gave *cis*- and *trans*-isomers, resulting in a poor harvest of the *trans*-isomer, so there is still an urgent need for further optimization and the development of novel methods to induce unidirectional conversion from the *cis*-isomer to the *trans*-isomer.

## Figures and Tables

**Table 1:** Different results caused by the catalysts and solvents used in condensation reaction.

Catalyst ^[a]^	Result	Solvent ^[b]^	Result
Sodium methoxide	Incomplete reaction	Ethanol	Favorable reaction
Sodium ethoxide	Incomplete reaction	Isopropanol	Favorable reaction
Potassium tert-butoxide	Favorable reaction	*tert*-Butanol	Favorable reaction
Sodium hydride	More by-products	*N*,*N*-dimethylformamide	Favorable reaction, but complicated work-up
*N*-butyl lithium	More by-products	Ether	Slow reaction
/	/	Tetrahydrofuran	More by-products

^[a]^ Potassium tert-butoxide was the best choice for the reaction. ^[b]^ The three alcohols exhibited good effects, and ethanol would be the first choice for large-scale production.

**Table 2:** Parameters for determination of anti-coccidial activity.

Group ^[#]^	RBWGR ^[a]^ (%)	SR ^[b]^ (%)	ALS ^[c]^	OS ^[d]^	ACI ^[e]^
I	100	100	0	0	200.00
II	46.02	40	32	20	34.02
III	99.68	100	0	1	198.68
IV	106.54	100	2	1	203.54
V	93.02	100	11	5	177.02

^[#]^ The chickens were housed for 20 days to get accustomed to the environment, and then divided into five groups. Group I (UI control) and Group II (I control) had daily access to standard chicken diet from day 0 to day 7. Group III (Hh6), Group IV (Hh3), and Group V (Hh1.5) were fed daily with the diet supplemented with halofuginone hydrobromide powder at the dose of 0.6% (6 mg/kg diet), 0.3% (3 mg/kg diet) or 0.15% (1.5 mg/kg diet), respectively. The initial number (n) of chickens in each group is indicated. ^[a]^ Relative Body weight gain rate (RBWGR); ^[b]^ Survival rate (SR); ^[c]^ Average lesion scores (ALS) ^[d]^ Oocyst score (OS); ^[e]^ Anticoccidial index (ACI): Halofuginone at doses of 0.6% and 0.3% exhibited high efficacy, in comparison, 0.15% exhibited moderate efficacy at best.
